# The cost of COVID-19 vaccine delivery in Bangladesh

**DOI:** 10.1080/21645515.2024.2411820

**Published:** 2024-10-18

**Authors:** Afroja Yesmin, Flavia Moi, Tarek Hossain, Rachel A. Archer, Monjurul Islam, Laura Boonstoppel

**Affiliations:** a ThinkWell, Dhaka, Bangladesh; b ThinkWell, Geneva, Switzerland; c ThinkWell, Manchester, UK; d Expanded Programme on Immunization (EPI), Directorate General of Health Services, Dhaka, Bangladesh

**Keywords:** COVID-19, vaccine, vaccination, immunization, costing, delivery, financing, Bangladesh

## Abstract

COVID-19 vaccination has been instrumental in fighting the pandemic, but evidence on the actual costs associated with delivering these vaccines in resource-constrained settings has been limited. We estimated the cost of delivering COVID-19 vaccines in Bangladesh through five delivery strategies in 2021 and 2022, including Ministry of Health (MOH) hospitals, non-MOH government hospitals, outreach at Expanded Program on Immunization (EPI) centers, mass campaigns, and schools. This was a bottom-up costing study, estimating costs from a payer and beneficiary perspective. We also mapped the funding flows for COVID-19 vaccination activities and analyzed programmatic and financial challenges. The economic cost incurred by the health system to deliver COVID-19 vaccines was $1.05 per dose, excluding vaccine costs. This was made up of a financial cost of $0.29 per dose and an opportunity cost of $0.75 per dose. School-based delivery incurred the lowest financial cost of $0.27, while outreach at EPI centers incurred the highest at $0.44 per dose. The low financial cost per dose is attributed to the high daily volumes delivered at sampled sites, minimal additional resources provided to sites to implement the COVID-19 vaccination program, and a reliance on the existing workforce. Beneficiaries spent an average of $1.63 to receive a single dose of COVID-19 vaccination at fixed sites, with transport representing the largest cost driver ($0.75 per dose). The economic cost to receive one dose of the COVID-19 vaccine was $4.78. Findings can support the Government of Bangladesh to make efficient and equitable resource allocation decisions for vaccination programs.

## Introduction

The COVID-19 pandemic led to profound health, social, and economic disruption.^1^ While COVID-19 vaccination has been pivotal in combating the global pandemic,^2^ delivering COVID-19 vaccines has been a monumental undertaking, particularly in low- and middle-income countries (LMICs) where resources are constrained. COVID-19 vaccination programs had to reach new target populations outside of the traditional routine immunization schedule, in enormous numbers. With the scale and complexity of COVID-19 vaccine delivery, there remained a clear need to understand the costs associated, in order to support the efficient and equitable resource management of the program, as well as future vaccine rollouts.

Prior research on the costs of delivering COVID-19 vaccines has been limited. At the time of the pandemic, in 2021, the COVAX Readiness and Delivery Working Group on Delivery Costing facility carried out global-level modeling of COVID-19 vaccine delivery costs and financing gaps for 133 LMICs.^3^ A study in Kenya derived a modeled estimation of the cost of delivering COVID-19 vaccines to different target groups in 2022.^4^ A significant evidence gap was left regarding the actual cost of delivering COVID-19 vaccines, and how this varied across delivery strategies and types of vaccination sites.

To bridge this gap, we estimated the cost of delivering COVID-19 vaccines across Bangladesh through five delivery strategies adopted by Government of Bangladesh (GOB) from a payer and beneficiary perspective. The study covers the period from the vaccine introduction in February 2021 until October 2022. We also examined how the COVID-19 vaccination program was implemented and financed.

### The COVID-19 vaccine roll-out in Bangladesh

The COVID-19 vaccination program in Bangladesh launched in February 2021, with the initial aim of vaccinating at least 80% of the total population.^5^ Due to supply constraints, the program initially prioritized frontline workers and individuals aged 60 and above for vaccination. When supply constraints eased in August 2021, the target population was expanded to all adults, and by October 2021, it was expanded further to include everyone over the age of 12. By June 2022, 83% of the total population had received two doses.^6^ In August 2022, vaccination opened to children aged 5–11 years. Coverage plateaued in 2023 as cases decreased, though when COVID-19 cases spiked in early 2024, a new campaign targeting priority groups was initiated.^7^

Under the guidance of the Prime Minister’s Office, the Directorate General of Health Services (DGHS) and the Expanded Programme on Immunization (EPI) headquarters executed the program, with support from implementing partners. The strategic direction was outlined in the National Deployment and Vaccination Plan,^8^ and various committees and working groups were established at national and sub-national levels to coordinate and implement the program. The vaccines and delivery costs were financed by the government budget, loans, and international donations.

Initially, COVID-19 vaccines were administered only at government hospitals. These were primarily Ministry of Health and Family Welfare (MOH) hospitals, though several other clinics within high-level government offices, and military-based hospitals, also functioned as fixed sites. As the program ramped up, COVID-19 vaccines were also delivered from EPI outreach centers, and temporary sites. Temporary sites were setup for mass vaccination campaigns, and at schools. In addition, occasionally, special campaigns for prisoners, vulnerable and floating populations were held. The COVID-19 vaccination rollout in Bangladesh was a remarkable achievement. In June 2022, the GOB achieved its aim of vaccinating 80% of the population against COVID-19, equating to 270 million COVID-19 vaccine doses.

## Methods

### Study design

This study retrospectively estimated the financial and economic costs of COVID-19 vaccine delivery at fixed and temporary sites in Bangladesh using a bottom-up (ingredients-based) approach. The methodology followed a general research protocol for a multi-country COVID-19 vaccine delivery costing project.^9^ The study estimated the costs from a payer and beneficiary perspective, the former encompassing costs incurred by the GOB and partners, and the latter capturing costs incurred by individuals to receive a single vaccine dose.

We captured recurrent costs, and start-up costs incurred by the health system in the 30 days before the study period until the end of the study period. Vaccine costs were excluded from the analysis due to sensitivities around these data. Costs were collected by program activity (such as service delivery, training, management, and reporting) and resource type (such as labor, allowances, fuel, and immunization supplies). All program activities and resource types are listed in Table 1 and defined in the Supplementary Material S1-S2. From beneficiaries, we collected demographic characteristics, income, time spent obtaining the COVID-19 vaccine, and direct financial expenses incurred. Key informant interviews and stakeholder consultation workshops were convened to determine the funding flows, and unpack the operational and financial challenges from implementation.

**Table 1.** Program activity and resource types.

Program activity types	Resource types
Program management; Vaccine collection, distribution and storage; Cold chain maintenance; Training; Social mobilization and advocacy; Supervision; Service delivery; Waste management; AEFI management; Record-keeping, HMIS, monitoring and evaluation	Paid labor, Volunteer labor; Honorarium; Per diem and travel allowances; Vaccine injection and safety supplies; Stationery and other supplies; Transport and fuel; Vehicle maintenance; Cold chain equipment repairs and energy costs; IEC and other printing costs; Workshops and meetings; Waste disposal fuel; Cold chain equipment; Vehicles; Incinerators

We estimated the cost of delivering COVID-19 through five delivery strategies in Bangladesh (see Table 2) from the start of the rollout in February 2021 until October 2022. Cost estimates reflect different time periods for each delivery strategy, based on when they were active.

**Table 2.** COVID-19 vaccine delivery strategies included in the study.

Delivery strategy	Time period	Definition	Site type
MOH hospitals	April-June 2022	Vaccination sites within health facilities, such as medical college hospitals, specialized hospital, district hospitals.	Fixed site
Non-MOH government hospitals	April-June 2022	Vaccination sites located at hospitals and clinics within high-level government offices such as defense forces hospitals, where COVID-19 vaccination was offered to government offices staff and dependents.	Fixed site
Outreach EPI centers	April-June 2022	EPI centers are similar to health posts or other fixed facilities, established by the EPI to extend immunization services in rural and urban areas, to reach communities who may not access regular health facilities.	Fixed site
Mass campaign	September-October 2022	Temporary vaccination sites established in urban and rural areas to bring vaccination closer to communities and reach wider coverage.	Temporary site
School-based	November 2021-March 2022	Vaccination sites occasionally set up in educational institutions, to reach students enrolled in schools.	Temporary site

### Sample

We employed a three-stage purposive sampling method to select vaccination sites across all eight divisions of Bangladesh. First, four city corporations and five districts were chosen in consultation with the EPI headquarters, to reflect varying parts of the country. Second, four upazilas from the selected districts were identified where the COVID-19 vaccination program was perceived as well-performing. Third, vaccination sites were chosen to represent different coverage levels and terrain (including hilly areas, islands, large cities, and hard-to-reach areas). Fixed sites were selected with input from the national-level EPI headquarters, while temporary sites were determined in collaboration with city corporation, civil surgeon, and upazila health offices.

The final sample covered 38 vaccination sites, of which 26 were fixed sites and 12 were temporary vaccination sites (see Table 3). 17 vaccination sites were in city corporations, 13 were in districts, and eight in upazilas. Out of the five non-MOH government hospitals in the sample, three were managed by the defense forces while the other two were hospitals for government employees. We also collected data from the national-level EPI headquarters, and the two main implementation partners – World Health Organization (WHO) and United Nations Children’s Fund (UNICEF).

**Table 3.** Study sample.

	Data collection sites				
	Fixed vaccination sites			Temporary vaccination sites	
	MOH hospitals	Non-MOH gov. hospitals	Outreach EPI centers	Mass campaign	School-based
City Corporation	5	4	2	2	4
District	5	1	3	2	2
Upazila	4		2	2	
Number of vaccination sites by strategy	14	5	7	6	6
Total vaccination sites	38				
National level: (EPI headquarters, UNICEF, WHO)	3				
Total data collection sites	41				

### Data collection

We collected data between September 2022 and March 2023. Cost data were collected from the sampled vaccination sites, the EPI headquarters, and partners through in-person interviews, document reviews, and inventory records, utilizing standardized data collection tools developed in Microsoft Excel. Beneficiary data were collected through 110 exit interviews conducted at six urban hospital-based sites. Informed consent was gathered from all participants.

We also carried out 53 key informant interviews, one at each of the 38 sampled vaccination sites and 15 at the national level. Key informants included health facility managers, focal persons for the vaccination sites, officials from EPI headquarters, DGHS, and MOH, as well as representatives from the Asian Development Bank, World Bank, UNICEF, WHO, Save the Children, and the Bangladesh Red Crescent Society. Five consultation workshops were convened at vaccination sites with relevant stakeholders supporting the program implementation. Workshops and interviews were recorded and subsequently transcribed.

### Data analysis

Costs were categorized as financial or opportunity costs and allocated to resource types, and program activities. We defined financial costs as direct financial expenses that were specifically related to the COVID-19 vaccination program, such as procurement of immunization supplies and new cold-chain equipment for the COVID-19 vaccination program, transport for COVID-19 vaccines, and salary for newly hired COVID-19 program personnel. Opportunity costs were defined as the value of using a share of existing resources for the COVID-19 vaccination program, such as labor of existing health staff and volunteers, or the value of using existing cold-chain equipment. While utilizing existing resources for the COVID-19 vaccine program does not require direct expenditures, allocating existing resources towards a new program leaves less resources available for other health services. Therefore, to show the total cost of delivering COVID-19 vaccines to the health system, we estimated the economic cost which includes both the opportunity cost and financial cost. Resources shared in the health system were allocated based on the proportion that resource was used for the COVID-19 vaccination program, as per respondent’s estimates. To calculate the opportunity cost or potential foregone income for beneficiaries, we valued time spent at the beneficiary’s income level. For individuals without formal employment, we used minimum wage to calculate the value of their time. Depreciation of capital items was calculated using the replacement price and useful life assumptions obtained from existing immunization costing guidance, with a discount rate of 3%.^10,11^

We estimated the unit cost per dose through a volume-weighted average approach:

$$\mathrm{unit}\_\cos t_{\mathrm{vw}}=\frac{\sum_{i=1}^{n}C_{i}}{\sum_{i=1}^{n}Q_{i}}$$

where Ci represents the total cost of vaccine delivery at location i, Qi is the total quantity of doses delivered at location i, and n is the sample size for that level. At the national level, we divided the total costs incurred by the total number of doses delivered in the same period. We then summed the volume-weighted average cost per dose for implementation level and the national level.

Costs are reported in 2022 US dollars (USD, $). We utilized the inflation rate (average consumer prices) published by the International Monetary Fund,^12^ to inflate costs incurred in 2021 to 2022. We converted costs from Bangladeshi Taka (BDT) to USD using a rate of 1 USD = 85.540939 BDT.^13^ When comparing our results to the available literature, we applied the same method to convert other studies’ findings into 2022 USD. Qualitative findings from the interviews were thematically analyzed.

## Ethical approval

Ethical approval for the study was obtained from the Institutional Review Board of the Institute of Health Economics (IHE-IRB), Federal-wide Assurance (FWA) No. FWA00026031. Administrative approval to conduct the study was granted by the Planning and Research Unit, DGHS and from the Public Health Wing, MOH.

## Results

### Descriptive statistics

We examined the days worked, doses delivered, number of staff and volunteers, and time spent on COVID-19 vaccination program activities, by delivery strategy, as shown in Table 4. In our sample, government hospitals delivered COVID-19 vaccines 6 days a week, while outreach at EPI centers did so 3 days a week. Mass campaign sites were active for 2 to 6 days at a time, while this significantly varied at school-based sites, between 1 to 181 days per site (see Table 4). The number of COVID-19 vaccine doses delivered per site per day was highest at school-based sites, which administered 983 doses on average. MOH hospitals also delivered very high volumes at 717 doses. Mass campaign sites delivered 320 doses per day/site and non-MOH government hospitals delivered 271 doses per day/site. Delivery volumes at EPI outreach centers were the lowest at 201 doses per day/site. EPI outreach centers spent the most time on vaccine administration per dose delivered (18 minutes) and schools spent the least (4 minutes). Despite delivery volumes varying substantially, for the other delivery strategies, time spent per dose delivered was relatively similar (between 5 and 9 minutes per dose) (Table 4).

**Table 4.** Average staffing and service delivery at the sampled vaccination sites (April-June 2022).

	Overall	Fixed sites			Temporary sites	
		MOH hospitals	Non-MOH gov. hospitals	Outreach EPI centers	Mass campaign sites	School-based
# vaccination sites	38	14	5	7	6	6
# vaccination days	–	6 days/week continuously		3 days/week continuously	2-6 days in total	1-181 days in total
Doses delivered per day	543	717	271	201	320	983
Vaccination team members	29	43	33	15	10	29
Of which regular staff	18	25	26	9	6	15
Of which volunteers	12	18	7	6	4	14
Person minutes (all activities)	22	13	22	55	17	12
Person minutes (vaccination)	8	5	9	18	6	4

We carried out exit interviews with 110 beneficiaries at fixed sites, comprising of 68 males and 42 females. Almost half of the respondents (51) reported not having any personal income, mostly homemakers and students. See Supplementary Material S3 for detail on beneficiaries’ demographic characteristics.

### Delivery cost per dose

The financial delivery cost ranged from $0.27 per dose at school-based sites, $0.29 at MOH hospitals, $0.33 at mass campaign sites, $0.36 at non-MOH government hospitals, and was highest at outreach EPI centers at $0.44 per dose (see Table 5). The financial delivery cost per dose was therefore inversely correlated with the number of doses that sites delivered on average per day. Allowances paid to volunteers was the largest financial delivery cost driver, representing between 31% and 62% of the cost per dose across all delivery strategies. Vaccine injection and safety supplies were also a financial cost driver, varying from $0.04–$0.06 (or 13% to 18%) across delivery strategies, as well as honorarium, a monetary incentive provided to the existing staff for working on the COVID-19 vaccination program. Across delivery strategies, the latter represented between $0.01 and $0.05 per dose (or 5% to 15%).

**Table 5.** Financial delivery cost per dose by delivery strategy broken down by resource type (2022 USD and %).

Resource types	Fixed site delivery			Temporary site delivery	
	MOH hospitals (717 doses/day)	Non-MOH government hospitals (271 doses/day)	Outreach EPI centers (201 doses/day)	Mass campaigns (320 doses/day)	School-based (983 doses/day)
Volunteer allowances	$0.15 (51%)	$0.20 (55%)	$0.23 (53%)	$0.10 (31%)	$0.16 (62%)
Vaccine injection and safety supplies	$0.04 (13%)	$0.05 (13%)	$0.06 (14%)	$0.05 (15%)	$0.05 (18%)
Honorarium	$0.04 (13%)	$0.05 (15%)	$0.04 (8%)	$0.04 (13%)	$0.01 (5%)
Transport and fuel	$0.02 (6%)	$0.01 (4%)	$0.02 (5%)	$0.03 (8%)	$0.01 (4%)
IEC and other printing costs	$0.01 (4%)	$0.01 (4%)	$0.02 (3%)	$0.02 (6%)	$0.01 (4%)
Per diem and travel allowances	$0.00 (0%)	$0.00 (0%)	$0.02 (6%)	$0.03 (8%)	$0.00 (1%)
Communication	$0.00 (0%)	$0.00 (0%)	$0.01 (2%)	$0.02 (6%)	$0.00 (0%)
Casual labor and national level new hires	$0.01 (5%)	$0.00 (0%)	$0.01 (1%)	$0.01 (2%)	$0.00 (0%)
Cold chain equipment	$0.00 (2%)	$0.01 (2%)	$0.00 (1%)	$0.00 (1%)	$0.01 (3%)
Refreshments	$0.00 (1%)	$0.01 (2%)	$0.00 (1%)	$0.02 (5%)	$0.00 (0%)
Waste collection & incinerators running costs	$0.00 (0%)	$0.00 (1%)	$0.00 (1%)	$0.00 (1%)	$0.00 (1%)
Workshops and meetings	$0.00 (1%)	$0.01 (2%)	$0.01 (3%)	$0.00 (0%)	$0.00 (0%)
Other costs*	$0.01 (3%)	$0.01 (2%)	$0.01 (2%)	$0.01 (3%)	$0.01 (2%)
**Total financial cost**	**$0.29**	**$0.36**	**$0.44**	**$0.33**	**$0.27**

*Includes cold chain repairs and energy costs, stationery and other supplies, costs related to the development of the registration app, vehicle maintenance, and vehicles.

**Figure 1.**
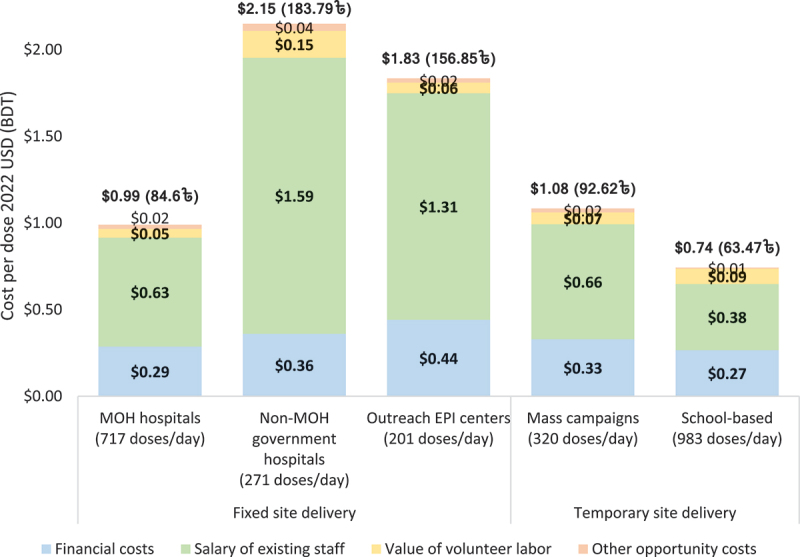
Economic delivery cost per dose by delivery strategy, with opportunity costs disaggregated by salary for existing staff, volunteer labor, and other opportunity costs (2022 USD, BDT).

The economic delivery cost per dose – which includes both financial and opportunity costs – varied much more across strategies, and ranged from $0.74 per dose at school-based sites, $0.99 at MOH hospitals, $1.08 at mass campaign sites, $1.83 at outreach EPI centers, to $2.15 per dose at non-MOH government hospitals (see Figure 1). Health worker salaries were the largest cost driver of the economic cost for all delivery strategies, making up at least half of the cost (50–74% across strategies). The average economic cost per dose for delivering COVID-19 vaccines across all delivery strategies was $1.05. Financial costs made up 27% of the total economic cost ($0.29), with opportunity cost accounting for the rest (73% or $0.75). See Supplementary Material S4-S5 for details.

Service delivery was the largest cost component for all types of sites except mass campaign sites, accounting for $0.25–$0.95 of the economic cost per dose (see Table 6). Program management was the biggest cost driver for mass campaign sites ($0.26 of the economic cost per dose). At mass campaign sites, program management was a labor-intensive activity with 40% of labor owed to program management. This proportion was much less for other delivery strategies at 4% to 16%. The second costliest activity at fixed sites was crowd controlling and client management (at $0.23-$0.59 per dose). Social mobilization costs were very low at fixed sites (at $0.01-$0.03 per dose). At temporary sites the cost structure differed slightly. Social mobilization comprised a larger share of the cost ($0.13-$0.14 per dose) and crowd controlling was a less significant cost component.

**Table 6.** Economic cost per dose by delivery strategy broken down by activity type (2022 USD and %).

Cost activities	Fixed site delivery			Temporary site delivery	
	MOH hospitals (717 doses/day)	Non-MOH government hospitals (271 doses/day)	Outreach EPI centers (201 doses/day)	Mass campaigns (320 doses/day)	School-based (983 doses/day)
Service delivery	$0.52 (52%)	$0.95 (44%)	$0.62 (34%)	$0.25 (23%)	$0.28 (37%)
Crowd controlling and client management	$0.23 (24%)	$0.59 (28%)	$0.32 (17%)	$0.14 (13%)	$0.13 (17%)
Vaccine distribution and storage	$0.07 (7%)	$0.16 (7%)	$0.13 (7%)	$0.11 (11%)	$0.03 (4%)
Supervision	$0.04 (4%)	$0.12 (6%)	$0.20 (11%)	$0.07 (7%)	$0.05 (6%)
Program management	$0.03 (3%)	$0.10 (5%)	$0.23 (13%)	$0.26 (24%)	$0.05 (7%)
Social mobilization	$0.01 (1%)	$0.03 (1%)	$0.03 (2%)	$0.13 (12%)	$0.14 (19%)
Record keeping, HMIS, M&E	$0.04 (4%)	$0.10 (5%)	$0.09 (5%)	$0.02 (2%)	$0.01 (1%)
Training	$0.01 (1%)	$0.03 (1%)	$0.15 (8%)	$0.02 (2%)	$0.01 (1%)
Waste management	$0.01 (1%)	$0.06 (3%)	$0.02 (1%)	$0.01 (1%)	$0.00 (1%)
AEFI management	$0.00 (0%)	$0.00 (0%)	$0.00 (0%)	$0.00 (0%)	$0.04 (5%)
Cold chain maintenance	$0.01 (1%)	$0.01 (0%)	$0.04 (2%)	$0.06 (5%)	$0.00 (1%)
**Total economic cost**	**$0.99**	**$2.15**	**$1.83**	**$1.08**	**$0.74**

### Beneficiary cost per dose

On average, the financial cost for beneficiaries to receive one dose of the COVID-19 vaccine was $1.63 at fixed sites. At $0.75 per dose (46% of the cost), transport costs to and from the vaccination site made up most of the cost. Beneficiaries spent $0.37 on registering for vaccination from an internet cafe and $0.35 on printing the vaccine certificate. Other direct financial expenses include costs related to managing side effects ($0.15) and food and drinks ($0.01) (detailed in Supplementary Material S6). The economic cost to receive one dose of the COVID-19 vaccine was $4.78, which includes financial costs along with the value of time spent obtaining the vaccination (on average 2 hours and 20 minutes), which was $3.14 per dose received. Value of time spent, representing potential lost income or the opportunity cost, reflected a median of 1% of beneficiaries’ monthly (personal or household) income. For just four (out of 89) beneficiaries that disclosed their household income, opportunity cost represented over 4% of their household’s income.

### Qualitative findings

**Figure 2.**
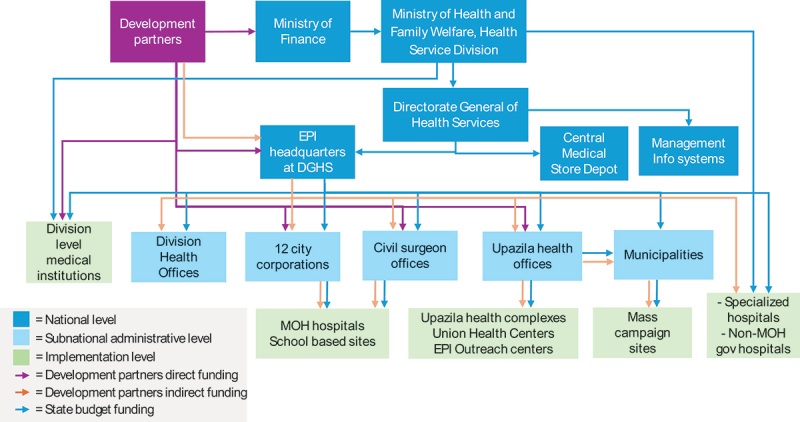
Fund flow for the C19 vaccination program.

From the qualitative assessment, a range of enablers and challenges in implementing the COVID-19 vaccination program, were obtained and summarized in Table 7. We also mapped the fund flow for the program in Figure 2.

**Table 7.** Enablers and challenges in implementing the COVID-19 vaccination program from key informants.

	Enablers	Challenges
Leadership	Robust political commitment, clear direction, and collaborative approach across government institutions. Regular supervision and direct support provided at implementation level.	(N/A)
Human resources	Strong sense of motivation and urgency among staff to make the program a success. Large numbers of volunteers mobilized thanks to the emergency context. Skilled and experienced personnel in the routine immunization program were leveraged to support COVID-19 vaccination.	Complex recruitment process meant that sites could not hire new staff to support COVID-19 vaccination. Program had to solely rely on the existing health workforce and volunteers. Human resource gaps, specifically at temporary sites, placed immense pressure on existing staff to meet the high demand for vaccination.
Financing	Timely financial and in-kind contributions from development partners for activities such as cold chain rentals and training. Off-budget financing mechanisms for partner funding helped facilitate activities that required sizable upfront investments, such as mass campaigns.	Limited funding at implementation level for essentials such as social mobilization and transport. Honorarium for staff discontinued, impeding staff morale and their commitment to the program. Rigid public financial management mechanisms hindered timely disbursement of government funds.
Supply and cold chain	(N/A)	Supply constraints when the program launched and short expiry dates for vaccines. Limited space and equipment to manage temperature-sensitive vaccines at the site level.
Social mobilization	Existing high levels of trust for the routine immunization program within communities.	(N/A)

## Discussion

This study examined the cost of delivering COVID-19 vaccines in Bangladesh through five delivery strategies in 2021 and 2022. We estimated the overall economic cost per dose incurred by the health system at $1.05, largely driven by the salaries of the existing health workforce. Existing resources within the EPI program were heavily leveraged to implement the program, as illustrated by the high opportunity cost in comparison with the financial cost found in this study ($0.29 across delivery strategies). The economic cost per dose estimate is lower than findings from prior immunization delivery costing studies in Bangladesh. Oral cholera vaccine (OCV) campaigns have been estimated to cost between $1.23 and $1.28 per dose (in 2022 USD).^14,15^ The lower cost in our study is likely due to the higher demand for COVID-19 vaccine and the high volumes delivered, though daily delivery volumes were not reported in these other studies.

At $0.29 per dose, Bangladesh incurred a very low financial cost in comparison to the other countries in the multi-country project which ranged from $0.43 in Mozambique,^16^ $0.60 in Vietnam,^17^ $0.67 in Côte d’Ivoire,^18^ $0.82 in Kampala (Uganda),^19^ $2.03 in Philippines,^20^ to $2.18 in the Democratic Republic of the Congo (DRC) (all in 2022 USD).^21^ We also observe a relatively lower economic cost per dose ($1.05) than most other study countries ranging from $1.78 in Vietnam,^17^ $2.43 in Kampala,^19^ $3.16 in Côte d’Ivoire,^18^ $3.58 in the Philippines,^20^ and $10.75 in the DRC,^21^ with the exception of Mozambique at $0.85^16^ (all in 2022 USD). This can also be put down to the sheer number of doses delivered per site in Bangladesh, which was much higher than other countries, and how the GOB quickly ramped up coverage at scale. By the end of the data collection period for fixed sites, Bangladesh had vaccinated 83% of total population, achieving a much higher coverage than other LMICs by that time.

The cost of COVID-19 vaccine delivery in our study is also much lower than the projections in the COVAX model for COVID-19 vaccine delivery in Bangladesh, estimated between $0.63 and $2.42 per dose.^3^ The model assumed that just 0–10% of the existing workforce would be redeployed for the program, with additional human resource needs met through hiring. In reality, Bangladesh hired no additional health workers for COVID-19 vaccination. Further, significant cost drivers in the COVAX model were per diems and travel costs, yet we found that these were relatively minimal on a per dose basis.

There is a dearth of available evidence on the cost beneficiaries incur to receive vaccination. A 2015 study in India, found the transport cost per individual to obtain OCV was only $0.02 per dose (2022 USD).^22^ This reflected vaccination being brought very close to households in this study (on average households were just 282.7 m from the nearest vaccination booth). On the contrary, our transport costs were estimated at $0.75 per dose and transport was our main cost driver, owed to the beneficiaries traveling to fixed sites. Another study in Guinea-Bissau found that mothers spent an average of $1.15 (2022 USD) on transport to access measles vaccination.^23^ This study was in a rural setting, where the vaccination sites were likely at a further distance from beneficiaries. Given the variability in findings among the limited number of studies on this topic, and the current focus on reaching “zero-dose” children with life-saving vaccines, more evidence is needed concerning the cost to receive vaccination through various delivery strategies.

Overall, the successful rollout, despite limited funding, can be attributed to strong political prioritization from the GOB, high level of dedication of staff and volunteers, being able to leverage resources from a high-performing national immunization program, and the large community trust in routine immunization. The estimated low financial cost per dose does not mean COVID-19 vaccination was a cheap program to deliver. Rather, this finding reflects no hiring of additional health workers, a reliance on existing infrastructure, and inadequate funding at implementation level. While the global community expected countries to mass hire to support the unprecedented scale of this vaccination effort in a global health emergency, Bangladesh relied exclusively on its existing workforce. Prior to the pandemic, Bangladesh was on the WHO health workforce support and safeguard list for critical staff shortages,^24^ and this persists post pandemic.^25^ In countries with a limited pool of human resources for conducing mass emergency vaccination drives, existing staff are diverted from providing other essential health services, leading to high opportunity costs. In this study, however, we did not quantify the impact of the COVID-19 vaccination program on the delivery of other health services. The emergency context meant that the health workforce and system were stretched beyond its limits to deliver in the short term, but long-term investment is needed to keep building a resilient primary health system in Bangladesh.

Lastly, we found that the most-efficient and equitable delivery strategies examined in our study were mass campaigns and school-based sites, which both brought vaccines close to communities, and accomplished high delivery volumes at low cost. In design, outreach EPI centers aim to reach vulnerable populations, making them the most equitable delivery strategy; however, we found the cost per dose to the health system highest at these centers. While the health-systems’ cost to deliver vaccines at MOH and non-MOH hospitals was low, beneficiaries incurred significant costs to access these fixed sites. Going forwards, cost, scale, and equity, of all strategies need to be factored into determining an optimal delivery strategy mix.

### Limitations

This study has several limitations. This study is based on the rollout of the COVID-19 vaccination in Bangladesh, delivered at an unprecedented scale during a global health pandemic. The results, therefore, are not representative of a routine situation and the results need to be considered in the context of an emergency. The cost estimates are based on a small sample, which may not represent all sites across the country. Owed to the purposive sampling approach, a disproportionate number of large hospitals were included in the sample, characterized by strong recordkeeping abilities and delivering doses in high numbers. Thus, the results might not fully represent smaller sites delivering low volumes of doses. Further, the COVID-19 vaccination program was performing well in all four upazilas identified for this study, the costs might have been higher at lower-performing upazilas. We only assessed beneficiary costs at fixed sites, and thus, only beneficiaries willing to travel to fixed sites were included in this study, potentially excluding less privileged individuals from the sample. These findings will likely be an overestimation of the cost incurred by beneficiaries accessing other sites, such as mass campaign sites. Our findings capture specific rollout periods, which may not be applicable to the recurrent costs during other program implementation phases. The study does not include contributions from the police force who provided security services and local government institutes who supported social mobilization, which may slightly underestimate the total vaccination program costs.

## Conclusion

This study provided evidence on the cost of delivering COVID-19 vaccines in Bangladesh during a pandemic, as well as an overview of programmatic and financial enablers and challenges identified during the roll-out. The findings can be used by the GOB and other countries to support planning and budgeting for their COVID-19 vaccination program and other vaccination programs. Political commitment from the GOB, coupled with exceptional dedication from health workers and communities, was the cornerstone to rapidly achieving high COVID-19 vaccination coverage in Bangladesh, with relatively limited funding. Elements of this study can also inform new vaccine instructions in the future, though a heavy reliance on existing resources is unsustainable in the long run, and it is highly unlikely the same enabling factors would hold true for future vaccine introductions.

## Data Availability

The data supporting this article is publicly available through the Harvard Dataverse at https://dataverse.harvard.edu/dataverse/thinkwell.
